# Hemogram responses in goats toward challenged with *Corynebacterium pseudotuberculosis* and its immunogen mycolic acids

**DOI:** 10.14202/vetworld.2017.655-661

**Published:** 2017-06-17

**Authors:** Mohammed Naji Odhah, Faez Firdaus Jesse Abdullah, Abd Wahid Haron, Mohd. Azmi Mohd. Lila, Mohd. Zamri-Saad, Zaid Khuder, Idris Umar Hambali, Muhammed Umar, Wessam Monther Saleh

**Affiliations:** 1 Department of Veterinary Clinical Studies, Faculty of Veterinary Medicine, Universiti Putra Malaysia, 43400 Serdang, Selangor, Malaysia; 2 Department of Veterinary Medicine, Faculty of Agriculture and Veterinary Medicine, Thamar University, Dhamar, Yemen; 3 Department of Veterinary Clinical Studies, Research Center for Ruminant Disease, Universiti Putra Malaysia 43400 Serdang Selangor, Malaysia; 4 Department of Veterinary Pathology and Microbiology, Faculty of Veterinary Medicine, Universiti Putra Malaysia, 43400 Serdang, Selangor, Malaysia; 5 Department of Veterinary Public Health and Preventive Medicine, University of Maiduguri, Nigeria; 6 Department of Animal Reproduction, Faculty of Verterinary and Animal Sciences, University of Agriculture, Water and Marine Sciences, Uthal, Baluchistan, Pakistan; 7 Department of Internal and Preventive Medicine, Faculty of Veterinary Medicine, University of Basra, Basra, Iraq

**Keywords:** complete blood count, *Corynebacterium pseudotuberculosis*, goat, hematology, mycolic acid

## Abstract

**Aim::**

This study was conducted to analyze the changes in blood profile of goats inoculated with *Corynebacterium pseudotuberculosis* and its immunogen mycolic acid (MA) extract.

**Materials and Methods::**

A total of 12 clinically healthy crossbred Boer female goats were divided into three groups; A, B and C (4 goats each per group). Group A was inoculated with 2 ml sterile phosphate buffered saline via intradermal route as the negative control group whilst Group B was inoculated with 2 ml of MA extract (1 g/ml) intradermally and Group C was then inoculated with 2 ml (1×10^9^) colony forming unit of active *C. pseudotuberculosis* intradermally. Blood sample was collected aseptically from the jugular vein periodically for complete blood count (CBC) analysis throughout the experimental period (3 months).

**Result::**

A significant decrease (p<0.05) was observed in red blood cells, hemoglobin (Hb), packed cell volume, mean corpuscular volume and mean corpuscular Hb concentration in Groups B and C as compared to the control while WBCs, neutrophil, lymphocyte and basophil showed a significant increase (p<0.05) as compared to the control.

**Conclusion::**

The inoculation of *C. pseudotuberculosis* and MA resulted in a significant change in the CBC, thereby, indicating that MA has a role in caseous lymphadenitis pathogenesis.

## Introduction

The alteration in blood during infectious diseases is discovered earlier in a hemogram and other blood biochemistry parameters which give an idea of the pathological conditions associated with the alterations. These alterations are as a result of the pathogen’s interaction with the immune components of the host [1,2]. The host immune components respond variably to different pathogens, pathogenesis, and inflammatory reactions. Acute bacterial infections are characterized by increased neutrophilic activity, while the chronic bacterial infections are characterized by lymphocytosis and monocytosis [3,4].

The incubation period of caseous lymphadenitis (CLA) ranges between 3 and 20 weeks, however, shorter incubation periods were reportedly associated [5,6] where few animals may likely develop clinical signs such as fever, changes in heart and respiratory rates, in appetence and decreased food consumption. CLA was reported not to have any significant changes on hemogram in goats that were challenged with *Corynebacterium pseudotuberculosis* bacterin, but a remarkable difference reflected significantly on the leukogram between the challenged groups at various sampling time [7-9]. Gameel and Tartour reported that sheep experimentally challenged with *C. pseudotuberculosis* bacteria showed alterations in the plasma proteins and the hemogram [10].

A report in 2016 indicated that serum amyloid A (SAA) had no response in relation to *C. pseudotuberculosis* and phospholipase D (PLD); however, haptoglobin did record a significant response to *C. pseudotuberculosis* and PLD [10]. The above report clearly infers that there is inverse relationship between *C. pseudotuberculosis*, PLD and acute phase protein concentration in goats. Therefore, future study on the effects of *C. pseudotuberculosis* and PLD on SAA will provide the gap of information [10].

In 2015, Mahmood *et al*. reported that *C. pseudotuberculosis* inoculations sequel into an appreciable response leading to changes in blood biochemistry, leukogram and hemogram [11]. The endotoxin (PLD) when inoculated also indicated a positive response in CLA pathogenesis; this further provides a window to better understanding of CLA pathogenesis and the major role PLD plays in the disease occurrence in goat with the various effects noticed in the blood biochemistry, hemogram and leukogram [12].

The cell walls of *Corynebacterium* spp. have a complex structure and are mostly considered a virulence factor in the occurrence of *C. pseudotuberculosis* [13,14]. The cell wall lipids signatures are 2-branched 3-hydroxy fatty acid which is usually named mycolic acid (MA) [15]. These buildings units in *C. pseudotuberculosis* expressed above are the main virulence factor found on the cell wall, and they contribute to its survival ability as a facultative intracellular parasite [16]. The most interesting aspect is that MA found in these bacteria exerts cytotoxic effects [17]. In several studies, it was shown that MA can be purified from *C. pseudotuberculosis* and inoculated in mice [18], the effects of which induces clinical signs such as congestion, hemorrhagic necrosis, and local edema [18]. *In-vitro* studies indicated that the phagocytic activity of WBC degenerated significantly during *C. pseudotuberculosis* infection [19] and that *C. pseudotuberculosis* bacterin resists cellular digestion by phagocytes [20], due to the subsequent development of abscesses. Many research have stated that the virulence factor of *C. pseudotuberculosis* is associated with the lipid contents of cell wall [21]. The information of hemogram profile in goats due to inoculation with MA extracted from *C. pseudotuberculosis* is scare. Therefore, the study aims to investigate and profile the hemogram responses in goats challenged with *C. pseudotuberculosis* and its immunogen MA.

## Materials and Methods

### Ethical approval

The experimental procedure was approved to be conducted under the Animal Care and Use Ethics Committee (IACUC No. R046/2015), Universiti Putra Malaysia (UPM) as required by the Animal Welfare Act (2015) in Malaysia.

### Bacteria and MA extraction

The *C. pseudotuberculosis* strain used in this study was originally isolated from chronic clinical case of CLA at TPU UPM goat farm [22]. The isolates were then sent to the Veterinary Laboratory Service Unit, Department of Veterinary Pathology and Microbiology at UPM for identification and confirmation. MA was extracted following the method described by Daffe and Etienne [23].

### Animals and experimental inoculations protocol

A total of 12 adult healthy crossbred Boer female goats aged between 16 and 20 months, and average weight of 20±5 kg were screened twice in the space of 3 months for CLA using the agar gel immune diffusion test before the onset of the actual experiment. The goats were housed in a facility isolated from the main faculty building. The house was fumigated and rested for 2 weeks before commencement of the experiments. The goats were randomly subdivided into three different groups consisting of four goats in each group similar to the procedure of Junior *et al*. [7] where the first group was inoculated with phosphate-buffered solution intradermally as the negative control (2 ml). The second group of goats was inoculated with 2 ml of MAs (1 mg/ml) intradermally, and third group goats were inoculated with (2 ml) *C. pseudotuberculosis* 1 × 10^9^ colony forming unit intradermally [11]. Blood collections were done via jugular vein at the first 24 h; thereafter, it continued for every 24 h post-inoculation for the first 7 days of the experiment. Subsequently, the blood collection was done twice a week up to 12 weeks post-inoculation. During the experimental procedure, feces and other waste products generated were regularly removed and disposed.

### Complete blood count (CBC)

Blood samples were analyzed using Animal Blood Counter (ABC*™*). The examined parameters were red blood cell (RBC), packed cell volume (PCV), hemoglobin (Hb), mean corpuscular volume (MCV), mean corpuscular Hb concentration (MCHC), neutrophils, lymphocytes, monocytes, basophils, eosinophil and total protein.

### Statistical analysis

Data obtained were analyzed using a statistical software JMP (version 9.0.1 SAS Institute Inc., Cary, NC, USA). Repeated measures ANOVA comparing values of treated groups and the control groups were conducted. All values obtained were reported as a mean±standard error and considered significant at p<0.05.

## Results

### CBC

The result for the complete blood count is as presented below.

#### RBCs count

There was significant decreased (p<0.05) in RBCs count in weeks 10 and 12 in Group 3 as compared to the Group A. While MA inoculated Group B showed no changes in the RBCs count (Table-1).

**Table-1:** RBCs response in female goat's after inoculation with *C. pseudotuberculosis* and MA (mean±SE).

Week	RBC count×10^12^/L		

	Group A	Group B	Group C
0	13.10±0.04^a^	13.07±0.04^a^	12.95±0.19^b^
1	12.80±0.16^b^	12.27±0.42^b^	11.70±0.27^bc^
2	13.15±0.15^a^	12.37±0.46^b^	11.90±1.17^bc^
3	12.17±0.70^b^	12.62±0.29^b^	11.72±0.95^bc^
4	12.92±0.04^b^	12.60±0.24^b^	13.05±0.06^a^
6	12.87±0.08^b^	12.50±0.41^b^	13.02±0.11^a^
8	13.07±0.04^a^	12.65±0.34^b^	12.27±0.16^b^
10	12.87±0.10^b^	12.75±0.22^b^	10.90±0.23^c^
12	12.17±0.70^a^	12.57±0.20^b^	10.72±1.36^c^

abc Values in different superscripts within row are significantly different at p<0.05, Group A: Buffered peptone saline, Group B: MA and Group C: *C. pseudotuberculosis.* *C. pseudotuberculosis*=*Corynebacterium pseudotuberculosis*, RBC=Red blood cell, MA=Mycolic acid, SE=Standard error

#### Concentration of Hb

The concentration of Hb indicated a significant decreased (p<0.05) in weeks 2, 3 and 12 in the Group C as compared to the control group and a significant decreased (p<0.05) in week 4 in Kindly provide text part Group B as compared with the control (Table-2).

**Table-2:** Hb concentration in female goat’s postinoculation with *C. pseudotuberculosis* and MA.

Weeks	Hb concentration g/L (Mean±SE)		

	Group A	Group B	Group C
0	93.6±3.95^a^	99.42±4.34^a^	99.55±2.16^a^
1	98.12±4.42^a^	93.47±1.12^ab^	91.50±2.06^bc^
2	95.07±3.31^ab^	91.90±1.57^bc^	72.27±10.39^c^
3	92.80±2.65^ab^	79.67±3.07^c^	73.27±9.82^c^
4	96.37±1.46^a^	74.10±4.50^c^	89.82±1.18^bc^
6	87.47±1.28^bc^	93.90±3.08^ab^	92.85±3.29^ab^
8	99.42±4.34^a^	91.10±4.36^bc^	83.00±2.58^bc^
10	93.60±3.95^ab^	93.10±3.29^ab^	82.75±2.71^bc^
12	92.80±2.65^ab^	94.97±3.75^ab^	73.95±9.40^c^

abc Values in different superscripts within row are significantly different at p<0.05. Group A: Buffered peptone saline, Group B: MA and Group C: *C. pseudotuberculosis. C. pseudotuberculosis*=*Corynebacterium pseudotuberculosis*, MA=Mycolic acid, SE=Standard error, Hb=Hemoglobin

#### The PCV

Group C indicated a significant increased (p<0.05) in weeks 3, 4 and 10 as compared with the control, while Group B showed a significant (p<0.05) increased in week 2 as compared with the control (Table-3).

**Table-3:** PCV in female goat’s postinoculation with *C. pseudotuberculosis* and MA.

Weeks	PCV L/L (Mean±SE)		

	Group A	Group B	Group C
0	0.20±0.00^b^	0.21±0.00^b^	0.21±0.00^b^
1	0.20±0.02^b^	0.21±0.00^b^	0.20±0.02^b^
2	0.21±0.00^b^	0.27±0.01^a^	0.21±0.01^b^
3	0.20±0.02^b^	0.20±0.02^b^	0.28±0.02^a^
4	0.22±0.01^b^	0.20±0.00^b^	0.27±0.01^a^
6	0.20±0.00^b^	0.22±0.02^b^	0.20±0.02^b^
8	0.21±0.03^b^	0.22±0.02^b^	0.21±0.01^b^
10	0.20±0.00^b^	0.20±0.00^b^	0.26±0.01^a^
12	0.22±0.02^b^	0.19±0.02^b^	0.20±0.01^b^

ab Values in different superscripts within row are significantly different at p<0.05. Group A: Buffered peptone saline, Group B: MA and Group C: *C. pseudotuberculosis. C. pseudotuberculosis*=*Corynebacterium pseudotuberculosis,* MA=Mycolic acid, SE=Standard error, PCV=Packed cell volume

#### MCV

Group C showed a significant increased (p<0.05) in weeks 2, 3 and 6 and a significant decreased (p<0.05) in weeks 10 and 12 compared to the control. Group B showed a significant increased (p<0.05) in weeks 4 and 12 compared to the control (Table-4).

**Table-4:** MCV in female goat’s postinoculation with *C. pseudotuberculosis* and MA.

Weeks	MCV fL (mean±SE)		

	Group A	Group B	Group C
0	21.25±0.62^ab^	20.50±0.62^b^	21.25±0.62^ab^
1	22.50±1.55^ab^	20.75±0.85^b^	22.25±0.47^ab^
2	20.50±0.86^b^	22.00±0.70^ab^	23.50±1.32^a^
3	20.25±0.62^b^	21.00±0.70^ab^	24.25±1.43^a^
4	19.75±0.47^bc^	23.00±2.04^a^	20.75±0.47^b^
6	21.00±0.40^ab^	22.75±1.81^ab^	23.00±0.40^a^
8	22.50±1.55^ab^	21.25±0.62^ab^	19.25±1.70^bc^
10	21.25±0.62^ab^	22.00±0.81^ab^	16.00±0.40^c^
12	22.25±1.03^ab^	23.00±0.91^a^	16.25±0.62^c^

abc Values in different superscripts within row are significantly different at p<0.05. Group A: Buffered peptone saline, Group B: MA and Group C: *C. pseudotuberculosis. C. pseudotuberculosis*=*Corynebacterium pseudotuberculosis*, MA=Mycolic acid, SE=Standard error, MCV=Mean corpuscular volume

#### MCHC

MCHC concentration was significantly decreased (p<0.05) in weeks 6, 10 and 12 in Group C as compared to the control, while Group B indicated a significant decreased (p<0.05) in week 8 as compared to the control group (Table-5).

**Table-5:** MCHC in female goat’s postinoculation with *C. pseudotuberculosis* and MA.

Weeks	MCHC fL (Mean±SE)		

	Group A	Group B	Group C
0	395.75±3.09^ab^	379.50±13.59^ab^	384.00±7.49^ab^
1	408.50±5.69^a^	379.75±13.88^ab^	375.75±10.22^ab^
2	416.75±14.48^a^	325.67±4.35^b^	348.50±23.25^b^
3	397.00±20.82^ab^	334.73±18.73^b^	368.25±14.00^b^
4	435.50±12.01^a^	337.22±8.55^db^	368.25±5.57^b^
6	378.75±12.57^ab^	334.87±5.56^b^	293.25±7.98^c^
8	381.50±10.71^ab^	297.85±2.38^c^	354.75±6.82^b^
10	419.00±16.33^a^	390.50±14.41^ab^	294.50±7.77^c^
12	386.75±11.19^ab^	377.50±8.76^ab^	292.50±14.70^c^

abc Values in different superscripts within row are significantly different at p<0.05. Group A: Buffered peptone saline, Group B: MA and Group C: *C. pseudotuberculosis. C. pseudotuberculosis*=*Corynebacterium pseudotuberculosis*, MA=Mycolic acid, SE=Standard error, MCHC=Mean corpuscular hemoglobin concentration

#### White blood cell count (WBC)

There was a significant increase in WBC count (p<0.05) in weeks 2, 3, 4 and 6 in Group C as compared to the control, and also a significant increased (p<0.05) in weeks 2 and 3 in Group B as compared to the control group (Table-6).

**Table-6:** WBC count in female goat’s postinoculation with *C. pseudotuberculosis* and MA.

Weeks	WBC count×10^9^/L (Mean±SE)		

	Group A	Group B	Group C
0	13.32±0.34^c^	13.97±0.95^c^	13.32±0.29^c^
1	12.52±0.43^cd^	14.97±1.54^c^	15.15±1.99^bc^
2	13.30±0.66^c^	18.30±1.98^ab^	19.82±0.57^ab^
3	11.22±1.96^d^	19.00±3.29^ab^	21.10±1.54^a^
4	11.57±0.73^d^	16.82±1.72^bc^	18.00±0.72^ab^
6	8.15±1.40^d^	15.57±0.68^bc^	18.60±0.97^ab^
8	12.07±0.27^cd^	16.30±2.20^bc^	14.42±1.80^c^
10	12.57±0.37^cd^	14.37±1.39^c^	15.37±1.88^bc^
12	11.22±0.92^cd^	12.27±1.84^cd^	15.42±0.95^bc^

abcd Values in different superscripts within row are significantly different at p<0.05. Group A: Buffered peptone saline, Group B: MA and Group C: *C. pseudotuberculosis*. WBC=White blood cell, *C. pseudotuberculosis*=*Corynebacterium pseudotuberculosis,* MA=Mycolic acid, SE=Standard error

Neutrophil count

The neutrophil count showed a significant increased (p<0.05) in weeks 2, 3 and 4 in Group C as compared to the control, and a significant increased (p<0.05) in week 4 for Group B as compared to the control group (Table-7).

**Table-7:** Neutrophil count response in female goat’s postinoculation with *C. pseudotuberculosis* and MA.

Weeks	Neutrophil count×10^9^/L (Mean±SE)		

	Group A	Group B	Group C
0	6.55±0.69^c^	7.58±1.22^bc^	8.03±1.86^ab^
1	7.90±1.21^bc^	8.62±2.41^ab^	9.13±1.29^ab^
2	7.03±0.43^bc^	9.17±1.56^ab^	14.18±0.82^a^
3	4.39±0.59^c^	13.94±1.38^a^	15.21±0.96^a^
4	5.40±0.56^c^	13.64±0.30^a^	12.18±0.89^a^
6	7.78±0.50^bc^	9.22±1.96^ab^	8.71±1.43^ab^
8	7.90±1.21^bc^	7.95±1.87^bc^	8.38±0.95^ab^
10	6.55±0.69^c^	8.45±1.56^ab^	8.95±1.12^ab^
12	4.39±0.59^c^	8.64±1.63^ab^	8.74±1.43^ab^

abc Values in different superscripts within row are significantly different at p<0.05. Group A: Buffered peptone saline, Group B: MA and Group C: *C. pseudotuberculosis. C. pseudotuberculosis*=*Corynebacterium pseudotuberculosis*, MA=Mycolic acid, SE=Standard error

Lymphocyte count

Group C showed a significant increased (p<0.05) in lymphocyte count for weeks 3, 4, 10 and 12 as compared to the control group, while Group B showed a significant increased (p<0.05) in weeks 2 and 3 as compared to the control group (Table-8).

**Table-8:** Lymphocyte count in female goat’s postinoculation with *C. pseudotuberculosis* and MA.

Weeks	Lymphocyte count×10^9^/L (Mean±SE)		

	Group A	Group B	Group C
0	3.59±0.27^c^	4.15±0.27^bc^	3.67±0.20^c^
1	5.12±0.99^b^	4.48±0.54^bc^	5.38±1.05^b^
2	4.75±0.79^bc^	8.04±0.69^a^	6.21±0.61^ab^
3	3.49±0.29^c^	8.31±1.13^a^	7.71±0.54^a^
4	4.60±0.72^bc^	6.70±0.92^ab^	7.84±0.81^a^
6	3.44±0.48^c^	6.97±0.80^ab^	6.71±0.89^ab^
8	3.62±0.66^c^	6.36±0.90^ab^	6.67±1.12^ab^
10	4.57±0.93^bc^	5.67±0.38^b^	7.23±0.92^a^
12	4.35±0.61^bc^	5.45±0.49^b^	7.12±0.21^a^

abc Values in different superscripts within row are significantly different at p<0.05. Group 1: Buffered peptone saline, Group 2: MA and Group 3: *C. pseudotuberculosis. C. pseudotuberculosis*=*Corynebacterium pseudotuberculosis,* MA=Mycolic acid, SE=Standard error

Monocyte count

Group C showed a significant increased (p<0.05) in weeks 2, 3, 4 and 6 as compared with the control, whereas monocyte count in Group B was significantly increased (p<0.05) at weeks 3, 4 and 10 as compared with the control and significantly decreased (p<0.05) in weeks 8, 10 and 12 for Group C and Group B in week 8 only as, respectively, compared with the control group (Table-9).

**Table-9:** Monocyte count in female goat’s postinoculation with *C. pseudotuberculosis* and MA.

Weeks	Monocyte count×10^9^/L (mean±SE)		

	Group A	Group B	Group C
0	0.66±0.03^c^	0.62±0.05^c^	0.62±0.01^c^
1	0.68±0.03^c^	0.71±0.11^b^	0.73±0.09^b^
2	0.71±0.02^b^	0.79±0.13^b^	1.34±0.09^a^
3	0.76±0.02^b^	1.11±0.10^a^	0.92±0.02^a^
4	0.67±0.01^c^	0.91±0.04^a^	0.92±0.08^a^
6	0.77±0.05^b^	0.79±0.09^b^	0.99±0.10^a^
8	0.74±0.11^b^	0.53±0.09^d^	0.62±0.09^c^
10	0.77±0.05^b^	0.93±0.15^a^	0.51±0.08^d^
12	0.68±0.05^c^	0.69±0.07^c^	0.46±0.04^d^

abcd Values in different superscripts within row are significantly different at p<0.05. Group A: Buffered peptone saline, Group B: MA and Group C: *C. pseudotuberculosis. C. pseudotuberculosis*=*Corynebacterium pseudotuberculosis*, MA=Mycolic acid, SE=Standard error

Basophil count

Group C showed a significant increased (p<0.05) of basophil count in weeks 3, 4, 6, 10 and 12 as compared to the control group, whereas Group B showed a significant increased (p<0.05) in weeks 2, 3, 4 and 8 as compared with the control group (Table-10).

**Table-10:** Basophil count in female goat’s postinoculation with *C. pseudotuberculosis* and MA.

Weeks	Basophil count×10^9^/L (Mean±SE)			

	Group A	Group B	Group C
0	0.11±0.01^b^	0.10±0.01^b^	0.09±0.00^b^
1	0.10±0.01^b^	0.16±0.02^ab^	0.15±0.02^ab^
2	0.12±0.02^b^	0.21±0.03^a^	0.15±0.01^ab^
3	0.15±0.02^ab^	0.24±0.02^a^	0.21±0.03^a^
4	0.09±0.01^b^	0.20±0.03^a^	0.25±0.04^a^
6	0.11±0.03^b^	0.18±0.02^ab^	0.24±0.06^a^
8	0.12±0.01^b^	0.26±0.06^a^	0.16±0.01^ab^
10	0.09±0.01^b^	0.18±0.03^ab^	0.16±0.02^ab^
12	0.15±0.05^ab^	0.18±0.07^ab^	0.20±0.00^a^

ab Values in different superscripts within row are significantly different at p<0.05. Group A: Buffered peptone saline, Group B: MA and Group C: *C. pseudotuberculosis. C. pseudotuberculosis*=*Corynebacterium pseudotuberculosis,* MA=Mycolic acid, SE=Standard error

Eosinophil count

Group C showed a significant increased (p<0.05) in eosinophilic count in weeks 2 and 4 with a significant decreased (p<0.05) in weeks 8 and 10 as compared with the control. Group B showed a significant increased (p<0.05) in weeks 2, 3 and 4 with a significant decreased (p<0.05) in weeks 8, 10 and 12 as compared to the control group (Table-11).

**Table-11:** Eosinophil count in female goat’s postinoculation with *C. pseudotuberculosis* and MA.

Weeks	Eosinophil count×10^9^/L (Mean±SE)		

	Group A	Group B	Group C
0	0.49±0.03^cd^	0.43±0.06^bc^	0.47±0.06^cd^
1	0.40±0.04^cd^	0.52±0.04^bc^	0.47±0.32^cd^
2	0.43±0.03^cd^	0.97±0.45^a^	0.61±0.06^b^
3	0.47±0.01^cd^	0.75±0.23^ab^	0.42±0.05^cd^
4	0.38±0.06^cd^	0.82±0.56^a^	0.59±0.06^b^
6	0.49±0.12^cd^	0.52±0.06^bc^	0.42±0.13^cd^
8	0.40±0.04^cd^	0.25±0.04^d^	0.26±0.08^d^
10	0.49±0.04^cd^	0.28±0.06^d^	0.23±0.07^d^
12	0.35±0.08^cd^	0.29±0.05^d^	0.35±0.07^cd^

abcd Values in different superscripts within row are significantly different at p<0.05. Group A: Buffered peptone saline, Group B: MA and Group C: *C. pseudotuberculosis. C. pseudotuberculosis*=*Corynebacterium pseudotuberculosis,* MA=Mycolic acid, SE=Standard error

### Total protein concentration

The total concentration of protein showed a significant increased (p<0.05) for Group C in week 1 but with a significant decreased (p<0.05) in weeks 8 and 10 as compared with the control group. However, Group B revealed a significant decreased (p<0.05) in weeks 2 and 10 as compared with the control group (Table-12).

**Table-12:** Total protein concentration in female goat’s postinoculation with *C. pseudotuberculosis* and MA.

Weeks	Total protein concentration g/L		

	Group A	Group B	Group C
0	76.00±5.22^ab^	79.25±4.78^ab^	80.50±2.63^ab^
1	71.00±5.58^b^	69.25±1.93^b^	90.25±2.78^a^
2	80.75±2.49^ab^	48.75±2.92^cd^	67.75±4.17^b^
3	73.25±3.19^b^	65.50±0.95^b^	70.75±2.21^b^
4	71.25±4.02^b^	67.50±0.64^b^	62.25±3.77^c^
6	70.25±2.71^b^	69.75±1.31^b^	65.50±3.09^b^
8	72.50±3.30^b^	72.25±1.65^b^	22.75±1.65^d^
10	78.00±2.79^ab^	49.75±4.85^cd^	39.75±1.10^d^
12	73.25±4.38^b^	68.75±1.54^b^	62.75±4.32^c^

abcd Values in different superscripts within row are significantly different at p<0.05. Group A: Buffered peptone saline, Group B: MA and Group C: *C. pseudotuberculosis. C. pseudotuberculosis*=*Corynebacterium pseudotuberculosis*, MA=Mycolic acid, SE=Standard error

## Discussion

The analysis of changes in blood via hematology evaluation indicated that it is of a great value in validating the predictive diagnosis of the disease. There is, however, a paucity of information on goat’s CBC analysis through the stages of *C. pseudotuberculosis* and MA. This study investigated the response of CBC to *C. pseudotuberculosis* and MA in goats after an experimental challenge.

This study reported and compared a wide range of hematological changes in the induced of CLA in goats and MA challenged goats. Kaplanski *et al*. [24] findings support our previous hypothesis that “MA inoculation led to significant increase in basophil count which interpreted as indicative of cellular immune response” a changing pattern of acute infection to chronic infection. There was an increased response in Group C (*C. pseudotuberculosis*) in relation to all the parameters measured, however, a lesser response was recorded in Group B (MA).

In this research, the hematological evaluation reached a point where it indicated the significant changes observed post-challenged with *C. pseudotuberculosis* and MA. Previous studies [7,11,25,26] reported quite similar findings to our present study where significant changes in RBC, MCHC, Hb concentration, and MCV were noticed. These results could be inferred as harmful effects of *C. pseudotuberculosis* and/or MA. Moreover, it was further hypothesized by Mahmood *et al*. [11], Osman *et al*. [25], and Russell *et al*. [27] that thrombocytopenia is a major cause of anemia or failure of megakaryocytes response in bone marrow. In this current investigation, there was striking difference where MA inoculated animals showed no significant changes in both RBC count and Hb concentrations. This very odd outcome might be an indicative of an insufficient dose of MA [28]. Moreover, there could be an association between MA and *C. pseudotuberculosis* as it relates to the occurrence of the disease, but the experiment group indicated that MA resulted in a classical CLA case with no alteration in the RBC and PCV counts [7]. The PCV showed a significant increase in both *C. pseudotuberculosis* and MA inoculated groups which disagree with the reports of Junior *et al.*, 2006. This might be due to with the direct effects of the MA or the indirect effects of the *C. pseudotuberculosis* causing degeneration of RBCs membrane [11,26,29]. Mahmood *et al*. [11] reported that *C. pseudotuberculosis* and PLD challenges have resulted into significant changes in the host hemogram, leukogram, and other blood biochemistry. Although the PLD plays an important role in CLA pathogenesis, yet when it is inoculated separately, it shows a varying response. In addition, factors such as age, sex, physiological status of the animal, and the individual variations may have an impact on the health status of the goats which in turn reflected in the blood screening [3,11]. There was an increased in WBC count in naturally infected CLA sheep due to the increased neutrophil, monocyte and lymphocyte counts [7,11,26]. Similarly, this study indicated that neutrophil, monocyte, and lymphocyte counts were significantly high when inoculated with the *C. pseudotuberculosis* and MA. These findings were in harmony with the reports from previous studies [11,25,26,30] where *C. pseudotuberculosis* infection resulted in a significant increase in most of the parameters. MA inoculation in this study resulted in transient immunosuppression which led to a significant rise in the neutrophil, monocyte and lymphocyte counts. MA has ability to attack. Consequently, activates neutrophil function during the course of vaccination and real exposure thereby led to an elevated response as seen in the previous studies carried out on related bacteria (*Mycobacterium bovis*) [15,31]. We hypothesized that MA from *C. pseudotuberculosis* behave in such a way similar to PLD in *M. bovis* causing a significant increase in neutrophil count in this present investigation, both the eosinophilic and basophilic counts were significantly increased in the challenged groups as well. These findings were not in agreement with reports by Ibtisam and Osman in 2008 and 2012 state that there was no significant change in basophilic count during CLA infection in sheep and mice. The finding of this study was in accord with Mahmood *et al*. [11] reported a significant increase in basophil count for *C. pseudotuberculosis* and PLD inoculated group of goats. Histologically, CLA abscessation in the lymph nodes of sheep and goats showed immense infiltration with neutrophil and to a lesser extent with eosinophils; these eosinophils give the push its greenish shade [32,33]. A study in 2015 Desvignes *et al*. suggested that basophil might be involved in cellular immunity as a regulator of T-cell in mediating the magnitude of the secondary immune response [34]. This study hypothesized that the significant rise in basophil count may due to the response of the cellular immunity toward MA immunogenic properties.

Furthermore, CLA affects the protein measure in the body [35] where the total body protein concentration is significantly altered post-inoculation with *C. pseudotuberculosis* and MA in this study. These findings disagree with the reports of Osman *et al*. [25] who indicated that the total protein concentration showed no significant changes in mice challenged with *C. pseudotuberculosis* and PLD. The results of this study are in agreement with the study carried out by Mahmood *et al*. [11]. The decreased in protein in CLA infected sheep might be associated with the liver damage in association with pathogen and its toxin. It could also be as a result of catabolism or the leakage of the plasma proteins into surrounding tissues since MA increases permeability of the capillaries [36,37]. There is dearth of on the effects of MA on total protein in goat.

## Conclusion

*C. pseudotuberculosis* and MA challenges have resulted in quite significant changes in the CBC as observed in this study. This provides a better understanding of the blood profile following CLA and the important role of MA in the disease occurrence. This study also reflects the role of MA in CLA as an indication of the pathological processes of the disease in female goat and its effects on various body systems and organs as it appeared in the CBC.

## Authors’ Contributions

FFJA and MAML, contributed to the conceptualization and design of this study and MNO ran the experiment and collected the samples, ZK and WMS assisted during the sample collection from goats, while AWH and MZS provided guidance during some aspect of laboratory experiments and in the statistical analysis of the results, IUH and MU assisted during animal study with feeding and cleaning of the pen. The authors also assisted in the development and editing of the manuscript before submission. Department of Veterinary Clinical Studies, Faculty of Veterinary Medicine, University Putra Malaysia, 43400 Serdang, Selangor, Malaysia. All authors read and approved the final manuscript.
